# Impact of COVID‐19 Restrictions on Estimating Causal Effects: Fluoroquinolones and Tendon Rupture

**DOI:** 10.1002/pds.70504

**Published:** 2026-10-15

**Authors:** Angel Y. S. Wong, Jeremy P. Brown, John Tazare, Marleen Bokern, Elizabeth Williamson, Jonathan Bartlett, Charlotte Warren‐Gash, Ian Douglas

**Affiliations:** ^1^ Faculty of Epidemiology and Population Health London School of Hygiene and Tropical Medicine London UK; ^2^ Faculty of Life Sciences & Medicine King's College London London UK

**Keywords:** causal inference, COVID‐19, healthcare delivery disruption, measurement error, real‐world data

## Abstract

**Purpose:**

The COVID‐19 pandemic disrupted healthcare services, potentially affecting causal effect estimation using real‐world data. As a case study, we replicated a cohort study using Clinical Practice Research Datalink Aurum to evaluate whether pandemic‐related disruptions influenced the measured association between fluoroquinolone use and tendon rupture.

**Methods:**

We included adults initiating fluoroquinolones/cephalosporins during 1 January 2006–31 March 2023. Four analyses compared treatment effect estimates using different periods for exposure and outcome ascertainment: pre‐pandemic only, pre‐pandemic exposure with follow‐up extending through the pandemic, all available data and post‐pandemic only. We estimated hazard ratios (HRs) for tendon rupture using Cox regression.

**Results:**

Among 891 968 fluoroquinolone users and 895 229 cephalosporin users, fluoroquinolone users had more comorbidities than cephalosporin users in the pre‐pandemic period but fewer in the post‐pandemic period. Pre‐pandemic analysis showed an increased risk of tendon rupture associated with fluoroquinolones (Propensity score weighted [PSW]‐HR: 1.94; 95% CI: 1.46–2.59), consistent with previous evidence. Estimates using all available data were similar for the overall study period (PSW‐HR: 1.91; 95% CI: 1.46–2.51) and the pre‐pandemic period (PSW‐HR: 1.96; 95% CI: 1.47–2.61) but were less precise during/post‐pandemic period (PSW‐HR: 1.57; 95% CI: 0.72–3.41) with no evidence of a period‐treatment interaction (PSW‐HR: 0.80; 95% CI: 0.35–1.84). Post‐pandemic analysis showed very few events among cephalosporin users but not fluoroquinolone users with a larger but imprecise estimate (PSW‐HR: 3.32; 95% CI: 0.62–17.72).

**Conclusion:**

In this case study, there was no clear evidence that pandemic‐related healthcare disruptions materially altered causal estimates, albeit with limited power to detect differences between before and during/post‐pandemic effects. Studies using real‐world data across the pandemic should routinely examine temporal changes in baseline characteristics and outcome incidence.

## Key Points

1. Combining data from pre‐, during and post‐pandemic periods produced relative and absolute effects that were broadly consistent with that from the pre‐pandemic period.
2. An inflated hazard ratio and wide confidence interval in the post‐pandemic analyses may reflect increased differential outcome ascertainment during/post‐pandemic or due to limited power for a rare outcome.
3. Future studies should report temporal trends of outcome incidences and baseline characteristics by exposure group.
4. A period–treatment interaction can help assess any differences in treatment effects between pre‐, during/post‐pandemic periods.
5. We recommend future research to investigate other types of outcomes and longer post‐pandemic periods.

## Plain Language Summary

The COVID‐19 pandemic changed healthcare access and how health information was recorded in electronic health records. These changes may affect study results that use routinely collected health data to assess safety and effectiveness of medications. We therefore examined whether the pandemic influenced measures of the association between fluoroquinolone and cephalosporin antibiotics and tendon rupture using UK primary care data.

We found that combining data collected before, during and post‐pandemic produced results that were similar to those using only pre‐pandemic data. This suggests that, in this example, pandemic‐related disruptions did not substantially change how we estimate medication effect on a rare outcome. Although the analyses using only post‐pandemic data showed a larger effect, the confidence intervals were very wide because relatively few tendon rupture outcomes occurred during/post‐pandemic. Differences in how outcomes were detected/recorded during this period may also have contributed.

Therefore, we recommend that future studies should report changes over time in patient characteristics and outcome rates for each treatment group to aid interpretation. Researchers should test whether treatment effects differ between pre‐ and during/post‐pandemic periods and investigate a wider range of outcomes using longer post‐pandemic follow‐up to better understand the long‐term impact of the pandemic on clinical studies.

## 1 Introduction

The COVID‐19 pandemic led to changes in health‐seeking behaviour and electronic health records (EHR) recording, which could lead to biases and hence invalid results, lessening the usefulness of EHR research covering this period of time [1].

In the United Kingdom, the COVID‐19 pandemic disrupted the National Health Service and also affected healthcare seeking behaviour, leading to considerable changes in recording of symptoms, healthcare contacts for preventive interventions, prescribing behaviour, drug stockpiling, treatment switching and diagnostic recording of many diseases in EHRs [1]. Our previous work using data from Clinical Practice Research Datalink (CPRD) showed substantial reductions in diagnoses for acute diseases following the introduction of lockdowns and restrictions since March 2020 [2]. Notably, this phenomenon of declines in disease diagnoses was not only observed in the UK data but also in many other countries [3, 4, 5, 6, 7, 8, 9, 10, 11, 12].

The impact of pandemic healthcare disruption on estimating causal effects has largely not been investigated empirically. Therefore, we replicated a published cohort study [13] as a case study to assess the potential impact of pandemic‐related healthcare disruptions (i.e., changes in health‐seeking behaviours and treatment decisions, and measurement errors) on estimating causal effects. In this case study, we estimated the effect of fluoroquinolones relative to cephalosporins on risk of tendon rupture, using data collected in pre‐, during and post‐pandemic periods, respectively. We chose this case study as tendon rupture is a well‐established adverse drug reaction [14] of fluoroquinolones. Second, people who would seek medical care in a non‐pandemic setting may not do so during a pandemic. As the outcome is a known consequence of fluoroquinolones but not cephalosporins, the likelihood of seeking medical attention for the outcome to be diagnosed and recorded may differ between fluoroquinolone and cephalosporin users with tendon rupture and it is therefore plausible for bias to occur when measuring treatment effects. Third, there has been good agreement in observational studies regarding the elevated risk, supported by plausible biological mechanism [14]. This presents the opportunity to use a recent cohort study [13] using the UK data to externally validate our findings. We therefore used this known effect to investigate the potential impact of measurement errors in outcome recording due to COVID‐19 pandemic lockdown on estimating causal effects for very rare outcomes.

## 2 Methods

### 2.1 Study Design

We replicated a cohort study using EHR data over different time periods to assess the potential impact of measurement errors due to pandemic lockdown on estimating the causal effect of fluoroquinolones on tendon rupture versus cephalosporins.

To assess the potential impact of pandemic‐related healthcare disruptions on causal effect estimation, we compared the treatment effect estimates in four analyses using different study periods to ascertain exposure and outcome (Figure 1): Analysis (1) using pre‐pandemic data only to ascertain both exposure and outcome, censoring all follow‐up at the start of the pandemic; Analysis (2) using pre‐pandemic data to ascertain exposure only, and then using pre‐, during and post‐pandemic data to ascertain outcome; Analysis (3) combining all data to ascertain exposure and outcome and Analysis (4) using post‐pandemic data only to ascertain exposure and outcome.

**FIGURE 1:**
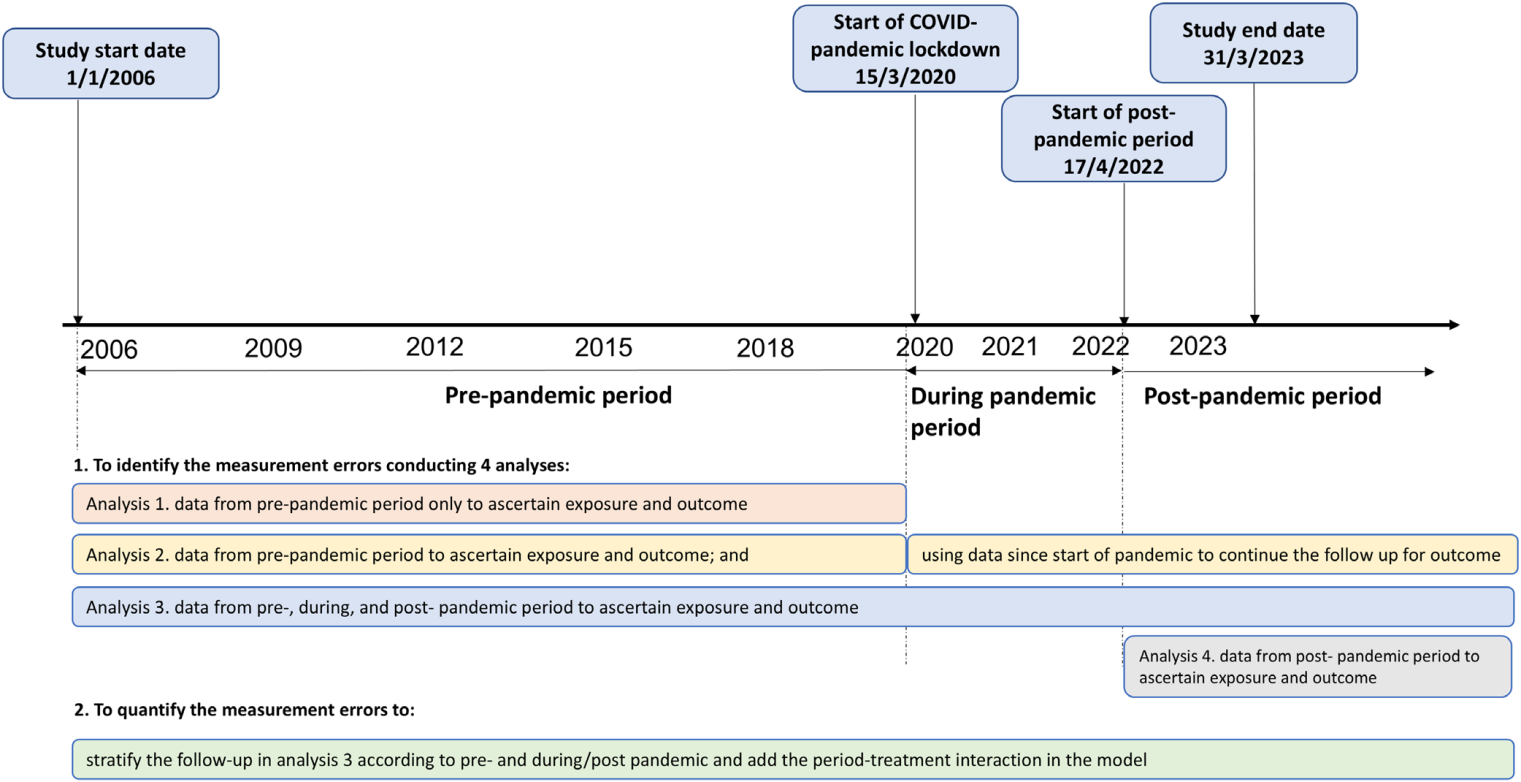
Illustration of four analyses using pre‐, during and post‐pandemic periods.

The start dates of the ‘during pandemic’ and the ‘post‐pandemic’ periods are informed by the COVID‐19 stringency index [15]. The start date of the ‘during pandemic’ period was 16 March 2020 when the UK government first recommended people to stop non‐essential contact and travel and the start of the ‘post‐pandemic’ period was 17 April 2022.

### 2.2 Data Source

We used data from the Clinical Practice Research Datalink (CPRD) Aurum. It contains primary care records of > 13 million currently registered patients from 1491 general practices in the United Kingdom. It is broadly representative in terms of age and sex of the general population [16]. We also used linked death data from the Office for National Statistics (ONS), hospital admissions data from Hospital Episode Statistics (HES) and individual‐level and practice‐level deprivation data from Index of Multiple Deprivation. As this study used routinely collected clinical data, patient consent is not required.

### 2.3 Study Population

We identified people aged ≥ 18 years with a first fluoroquinolone or cephalosporin prescription during the total study period of 1 January 2006–31 March 2023. We ended the study period on 31 March 2023 as it was the last date of the latest linked HES data availability. We only included those with linkage to HES/ONS and who had at least 1‐year prior registration of patients at practice or practice data deemed by the data provider to be of research quality. Notably, these database specific inclusion criteria would not be impacted by the pandemic. People who had a diagnosis of tendon rupture within 1 year before cohort entry were excluded.

### 2.4 Exposure

The exposed group was those who received their first systemic fluoroquinolone prescription (ciprofloxacin, levofloxacin, moxifloxacin, norfloxacin or ofloxacin) while the comparison group was those who received their first systemic cephalosporin prescription in the total study period of 1 January 2006–31 March 2023. In the comparison group, people who received fluoroquinolones within 60 days before their cohort entry date were excluded. We chose cephalosporins as they have a similar prescribing profile in the UK practice to fluoroquinolones and are not known to have a causal effect on tendon rupture [17].

### 2.5 Outcomes

The outcome was a patient's first diagnostic record of tendon rupture during their follow‐up. As in the previous study [13], we used pre‐specified diagnostic codelists to ascertain the outcome using data from CPRD and HES, recorded by GPs and clinicians responsible for their hospital episodes of care, and did not necessarily represent diagnoses made by a specialist. We started the follow‐up at the initiation of fluoroquinolones or cephalosporin, and it ended at the earliest of a diagnosis of tendon rupture, study end date (varied across different analyses), switching to comparator antibiotic, patient transferred out of practice date, date of last data collection at practice and patient death or 60 days following antibiotic prescription. Figure S1 illustrates the follow‐up time.

### 2.6 Covariates

Covariates were potential confounders between prescribing fluoroquinolones (as opposed to cephalosporin) and tendon rupture. These were selected based on clinical knowledge and informed by a previous cohort study [13]. Included confounders were age, sex, deprivation, region, body mass index, smoking status, alcohol consumption, ethnicity, number of hospital admissions, number of active GP visits, prior comorbidities (hypertension, diabetes, uncontrolled diabetes, end‐stage renal disease, multiple sclerosis, rheumatoid arthritis, trauma/injuries in the past 90 days, osteoarthritis, gout, lupus, parathyroid disorders, thyroid disorders) and corticosteroid use and bisphosphonate use in 6 months prior.

### 2.7 Statistical Analyses

We reported the incidence of tendon rupture by exposure group from 1 January 2006 to 31 December 2022. In each analysis based on different study periods, we used propensity scores (PS) to re‐weight the sample to achieve balance between exposure groups conditional on observed baseline confounders [18]. We derived PS from a logistic regression model where the dependent variable is prescription of fluoroquinolone (as opposed to cephalosporin), including the potential confounders as covariates, and used the PS to create inverse probability‐of‐treatment weights separately in each analysis. The PS weights were calculated based on PS distribution in all patients for obtaining an average treatment effect. We restricted the cohort to those individuals whose PS values were within the overlapping region of the distributions of the fluoroquinolone group and the cephalosporin group [19]. We estimated hazard ratios (HRs) by comparing the hazards of tendon rupture for the fluoroquinolone group with the cephalosporin group using stabilised inverse probability‐of‐treatment‐weighted Cox regression with robust standard errors. Time since the start of prescription was the timescale. We also estimated the crude and PS‐weighted rate difference (RD) for tendon rupture between fluoroquinolone and cephalosporin users using Poisson regression with robust standard errors in each analysis. We accounted for the competing risk of death by censoring individuals who died before having tendon rupture. We used complete case analysis to address missing data. In Analysis 3 (Figure 1), we allowed treatment effects to vary across time periods (pre‐, and during/post‐pandemic periods) by including the period as a time‐dependent covariate and a period‐treatment interaction in the outcome model. Supporting Information S1 shows details of implementation of period‐treatment interaction.

For external validation, we assessed whether our result in Analysis 1 was consistent with the previous cohort study [13] based on two pre‐specified criteria. First, the effect size must be clinically comparable with previous findings meaning the point estimate in our study must lie within the confidence intervals of the previous findings (i.e., propensity score weighted HR: 1.89; 95% CI: 1.45–2.46). Second, the 95% confidence interval must exclude 1, as in the previous study.

We assessed the proportions of people deregistered from their GP and switching to another treatment group respectively in each analysis to evaluate if there was differential loss to follow‐up across time periods. We also compared baseline characteristics of the study cohort in pre‐, during/post and post‐pandemic periods respectively, which allowed evaluation of the impact of the pandemic on study cohort inclusion.

### 2.8 Sensitivity Analyses

Since primary care data was available for a longer time period than hospital data, we assessed sensitivity of results to a longer post‐pandemic period by extending the end of study period to 31 December 2024 and using primary care data only to ascertain covariates and outcomes. This increased data from the post‐pandemic period to inform how any changes in diagnostic recording affected the estimation of causal effects in the longer term. Second, we varied the study start year from 2006 to 2019 to examine the impact of including different proportions of study time covered by the pandemic.

Stata/MP 19 was used for data processing and analyses. Codelists and analytical codes are available in: https://doi.org/10.17037/DATA.00005295 and https://github.com/ehr‐lshtm/covid‐causal‐inference‐FQ. ChatGPT was used to improve the clarity and readability of the English in the manuscript.

## 3 Results

We identified 891 968 eligible fluoroquinolone users and 895 229 eligible cephalosporin users during 1 January 2006–31 March 2023 (Figure S2). Generally, we observed a decreasing trend of prescribing fluoroquinolones and cephalosporin from 2006 to 2022, but prescribing of cephalosporin slightly increased from 2020 to 2022. Before COVID‐19 pandemic lockdown, fluoroquinolone users were more likely to be older, male, white, current alcohol drinkers, less deprived, registered with a GP in the West Midlands and Southwest and to have had ≥ 12 GP active consultations and hospital admissions in the past year, compared with cephalosporin users. Both exposure groups had comparable distributions of medical conditions and medication use, but fluoroquinolone users had marginally greater comorbidities and were more likely to be prescribed corticosteroid than cephalosporin users (Table 1).

**TABLE 1:** Baseline characteristics of the study cohort.

Study cohort	Pre‐pandemic (1 January 2006–15 March 2020) (Analysis 1 or Analysis 2)		Pre, during, post‐pandemic (1 January 2006–31 March 2023) (Analysis 3)		During/post‐pandemic (16 March 2020–31 March 2023)		Post‐pandemic (17 April 2022–31 March 2023) (Analysis 4)	
	Cephalosporin	Fluoroquinolone	Cephalosporin	Fluoroquinolone	Cephalosporin	Fluoroquinolone	Cephalosporin	Fluoroquinolone
Total	818 669	798 422	895 229	891 968	76 558	93 544	26 319	28 255
Age at index date								
Median (IQR)	53.6 (35.2–72.3)	55.7 (39.6–71.1)	53.7 (35.2–72.5)	55.5 (39.3–71.1)	54.7 (34.6–74.5)	54.2 (37.3–70.6)	54.6 (34.6–74.0)	54.5 (37.4–70.7)
Min, Max	18.0, 111.5	18.0, 110.9	18.0, 111.5	18.0, 110.9	18.0, 108.3	18.0, 108.4	18.0, 106.3	18.0, 107.1
Age group, *N* (%)								
18 to < 40	265 274 (32.4)	204 975 (25.7)	290 795 (32.5)	232 216 (26.0)	25 520 (33.3)	27 241 (29.1)	8786 (33.4)	8150 (28.8)
40 to < 50	108 092 (13.2)	124 202 (15.6)	116 457 (13.0)	137 538 (15.4)	8364 (10.9)	13 336 (14.3)	2848 (10.8)	4000 (14.2)
50 to < 60	102 600 (12.5)	123 983 (15.5)	112 105 (12.5)	139 151 (15.6)	9505 (12.4)	15 168 (16.2)	3416 (13.0)	4538 (16.1)
60 to < 70	112 558 (13.7)	131 284 (16.4)	121 842 (13.6)	144 911 (16.2)	9284 (12.1)	13 625 (14.6)	3309 (12.6)	4221 (14.9)
70 to < 80	111 856 (13.7)	116 223 (14.6)	122 796 (13.7)	129 630 (14.5)	10 940 (14.3)	13 407 (14.3)	3796 (14.4)	4115 (14.6)
80+	118 289 (14.4)	97 755 (12.2)	131 234 (14.7)	108 522 (12.2)	12 945 (16.9)	10 767 (11.5)	4164 (15.8)	3231 (11.4)
Male sex, *N* (%)	244 475 (29.9)	411 755 (51.6)	264 302 (29.5)	466 744 (52.3)	19 827 (25.9)	54 988 (58.8)	6860 (26.1)	16 589 (58.7)
Calendar year at cohort entry, *N* (%)								
2006	134 715 (16.5)	86 397 (10.8)	134 715 (15.0)	86 397 (9.7)	NA	NA	NA	NA
2007	133 174 (16.3)	90 137 (11.3)	133 174 (14.9)	90 137 (10.1)	NA	NA	NA	NA
2008	106 319 (13.0)	75 326 (9.4)	106 319 (11.9)	75 326 (8.4)	NA	NA	NA	NA
2009	80 349 (9.8)	65 448 (8.2)	80 349 (9.0)	65 448 (7.3)	NA	NA	NA	NA
2010	69 362 (8.5)	60 324 (7.6)	69 362 (7.7)	60 324 (6.8)	NA	NA	NA	NA
2011	53 946 (6.6)	54 549 (6.8)	53 946 (6.0)	54 549 (6.1)	NA	NA	NA	NA
2012	44 344 (5.4)	53 763 (6.7)	44 344 (5.0)	53 763 (6.0)	NA	NA	NA	NA
2013	39 497 (4.8)	49 297 (6.2)	39 497 (4.4)	49 297 (5.5)	NA	NA	NA	NA
2014	36 784 (4.5)	47 704 (6.0)	36 784 (4.1)	47 704 (5.3)	NA	NA	NA	NA
2015	29 371 (3.6)	43 920 (5.5)	29 371 (3.3)	43 920 (4.9)	NA	NA	NA	NA
2016	24 856 (3.0)	42 397 (5.3)	24 856 (2.8)	42 397 (4.8)	NA	NA	NA	NA
2017	21 604 (2.6)	42 097 (5.3)	21 604 (2.4)	42 097 (4.7)	NA	NA	NA	NA
2018	20 031 (2.4)	43 448 (5.4)	20 031 (2.2)	43 448 (4.9)	NA	NA	NA	NA
2019	20 063 (2.5)	36 628 (4.6)	20 063 (2.2)	36 628 (4.1)	NA	NA	NA	NA
2020	4254 (0.5)	6987 (0.9)	22 479 (2.5)	32 844 (3.7)	18 223 (23.8)	25 855 (27.6)	NA	NA
2021	NA	NA	24 550 (2.7)	30 936 (3.5)	24 550 (32.1)	30 936 (33.1)	NA	NA
2022	NA	NA	26 561 (3.0)	28 926 (3.2)	26 561 (34.7)	28 926 (30.9)	19 095 (72.6)	20 428 (72.3)
2023*	NA	NA	7224 (0.8)	7827 (0.9)	7224 (9.4)	7827 (8.4)	7224 (27.4)	7827 (27.7)
Body mass index, *N* (%)								
Underweight	24 926 (3.0)	19 749 (2.5)	27 477 (3.1)	22 204 (2.5)	2551 (3.3)	2455 (2.6)	883 (3.4)	785 (2.8)
Normal	298 579 (36.5)	267 879 (33.6)	324 811 (36.3)	297 032 (33.3)	26 232 (34.3)	29 152 (31.2)	8957 (34.0)	8647 (30.6)
Overweight	248 757 (30.4)	263 785 (33.0)	271 083 (30.3)	293 652 (32.9)	22 325 (29.2)	29 867 (31.9)	7570 (28.8)	8969 (31.7)
Obese	197 495 (24.1)	197 843 (24.8)	219 665 (24.5)	223 488 (25.1)	22 169 (29.0)	25 644 (27.4)	7691 (29.2)	7769 (27.5)
Missing	48 912 (6.0)	49 166 (6.2)	52 193 (5.8)	55 592 (6.2)	3281 (4.3)	6426 (6.9)	1218 (4.6)	2085 (7.4)
Smoking status, *N* (%)								
Non‐smoker	281 369 (34.4)	250 212 (31.3)	303 737 (33.9)	275 916 (30.9)	22 368 (29.2)	25 704 (27.5)	7620 (29.0)	7633 (27.0)
Current smoker	246 225 (30.1)	240 954 (30.2)	265 044 (29.6)	266 022 (29.8)	18 818 (24.6)	25 068 (26.8)	6346 (24.1)	7327 (25.9)
Ex‐smoker	288 854 (35.3)	304 656 (38.2)	323 509 (36.1)	346 163 (38.8)	34 654 (45.3)	41 505 (44.4)	12 034 (45.7)	12 810 (45.3)
Missing	2221 (0.3)	2600 (0.3)	2939 (0.3)	3867 (0.4)	718 (0.9)	1267 (1.4)	319 (1.2)	485 (1.7)
Ethnicity, *N* (%)								
White	707 746 (86.5)	709 832 (88.9)	773 326 (86.4)	791 098 (88.7)	65 579 (85.7)	81 264 (86.9)	22 351 (84.9)	24 393 (86.3)
South Asian	50 895 (6.2)	37 556 (4.7)	56 164 (6.3)	42 771 (4.8)	5268 (6.9)	5215 (5.6)	1890 (7.2)	1719 (6.1)
Black	27 074 (3.3)	19 858 (2.5)	29 493 (3.3)	22 925 (2.6)	2419 (3.2)	3067 (3.3)	851 (3.2)	903 (3.2)
Other	10 926 (1.3)	8697 (1.1)	12 610 (1.4)	10 372 (1.2)	1684 (2.2)	1675 (1.8)	640 (2.4)	574 (2.0)
Mixed	7183 (0.9)	5913 (0.7)	8223 (0.9)	7058 (0.8)	1040 (1.4)	1145 (1.2)	363 (1.4)	340 (1.2)
Not stated	7491 (0.9)	7681 (1.0)	7887 (0.9)	8423 (0.9)	396 (0.5)	742 (0.8)	163 (0.6)	215 (0.8)
Missing	7354 (0.9)	8885 (1.1)	7526 (0.8)	9321 (1.0)	172 (0.2)	436 (0.5)	61 (0.2)	111 (0.4)
Index of multiple deprivation, *N* (%)								
1 (least deprived)	159 416 (19.5)	178 483 (22.4)	174 648 (19.5)	198 852 (22.3)	15 232 (19.9)	20 368 (21.8)	5355 (20.3)	6109 (21.6)
2	161 518 (19.7)	171 440 (21.5)	176 758 (19.7)	191 063 (21.4)	15 240 (19.9)	19 622 (21.0)	5182 (19.7)	5880 (20.8)
3	155 110 (18.9)	155 933 (19.5)	169 679 (19.0)	174 164 (19.5)	14 569 (19.0)	18 231 (19.5)	4940 (18.8)	5483 (19.4)
4	170 604 (20.8)	153 178 (19.2)	186 444 (20.8)	171 375 (19.2)	15 840 (20.7)	18 197 (19.5)	5419 (20.6)	5522 (19.5)
5 (most deprived)	172 021 (21.0)	139 388 (17.5)	187 700 (21.0)	156 514 (17.5)	15 677 (20.5)	17 126 (18.3)	5423 (20.6)	5261 (18.6)
Alcohol status, *N* (%)								
Non‐drinker	83 761 (10.2)	62 887 (7.9)	91 893 (10.3)	70 521 (7.9)	8132 (10.6)	7634 (8.2)	2845 (10.8)	2335 (8.3)
Current low level	367 206 (44.9)	366 915 (46.0)	396 775 (44.3)	404 923 (45.4)	29 568 (38.6)	38 008 (40.6)	9931 (37.7)	11 268 (39.9)
Current Medium level	56 359 (6.9)	81 425 (10.2)	60 285 (6.7)	89 526 (10.0)	3926 (5.1)	8100 (8.7)	1365 (5.2)	2356 (8.3)
Current high level	29 213 (3.6)	37 920 (4.7)	33 129 (3.7)	44 125 (4.9)	3916 (5.1)	6205 (6.6)	1323 (5.0)	1814 (6.4)
Current drinker (missing: consumption level)	116 399 (14.2)	98 156 (12.3)	126 659 (14.1)	108 930 (12.2)	10 260 (13.4)	10 773 (11.5)	3307 (12.6)	2962 (10.5)
Ex‐drinker	96 732 (11.8)	84 775 (10.6)	110 244 (12.3)	98 429 (11.0)	13 511 (17.6)	13 654 (14.6)	4911 (18.7)	4524 (16.0)
Missing	68 999 (8.4)	66 344 (8.3)	76 244 (8.5)	75 514 (8.5)	7245 (9.5)	9170 (9.8)	2637 (10.0)	2996 (10.6)
Region, *N* (%)								
North East	28 177 (3.4)	18 074 (2.3)	30 095 (3.4)	20 399 (2.3)	1918 (2.5)	2325 (2.5)	604 (2.3)	681 (2.4)
North West	197 572 (24.1)	154 155 (19.3)	216 277 (24.2)	171 491 (19.2)	18 704 (24.4)	17 335 (18.5)	6502 (24.7)	5311 (18.8)
Yorkshire and The Humber	31 534 (3.9)	21 377 (2.7)	33 529 (3.7)	23 559 (2.6)	1995 (2.6)	2182 (2.3)	574 (2.2)	569 (2.0)
East Midlands	18 425 (2.3)	18 069 (2.3)	20 633 (2.3)	19 757 (2.2)	2208 (2.9)	1688 (1.8)	695 (2.6)	447 (1.6)
West Midlands	104 344 (12.7)	124 682 (15.6)	113 598 (12.7)	142 562 (16.0)	9254 (12.1)	17 880 (19.1)	3388 (12.9)	5586 (19.8)
East of England	33 699 (4.1)	36 907 (4.6)	37 505 (4.2)	41 234 (4.6)	3806 (5.0)	4327 (4.6)	1425 (5.4)	1423 (5.0)
London	152 914 (18.7)	131 756 (16.5)	166 378 (18.6)	147 220 (16.5)	13 464 (17.6)	15 464 (16.5)	4490 (17.1)	4768 (16.9)
South East	175 023 (21.4)	180 642 (22.6)	191 228 (21.4)	201 976 (22.6)	16 204 (21.2)	21 333 (22.8)	5574 (21.2)	6273 (22.2)
South West	76 981 (9.4)	112 760 (14.1)	85 986 (9.6)	123 770 (13.9)	9005 (11.8)	11 010 (11.8)	3067 (11.7)	3197 (11.3)
Medical history, *N* (%)								
Hypertension	242 789 (29.7)	247 365 (31.0)	268 709 (30.0)	277 097 (31.1)	25 920 (33.9)	29 730 (31.8)	8804 (33.5)	9092 (32.2)
Diabetes	95 045 (11.6)	100 749 (12.6)	110 611 (12.4)	118 146 (13.2)	15 566 (20.3)	17 396 (18.6)	5668 (21.5)	5664 (20.0)
Uncontrolled diabetes	48 841 (6.0)	54 225 (6.8)	55 755 (6.2)	62 301 (7.0)	6914 (9.0)	8075 (8.6)	2398 (9.1)	2532 (9.0)
Multiple sclerosis	4234 (0.5)	4356 (0.5)	4701 (0.5)	4888 (0.5)	467 (0.6)	532 (0.6)	153 (0.6)	145 (0.5)
Rheumatoid arthritis	13 706 (1.7)	12 040 (1.5)	15 285 (1.7)	13 686 (1.5)	1579 (2.1)	1645 (1.8)	588 (2.2)	514 (1.8)
Osteoarthritis	158 029 (19.3)	162 535 (20.4)	176 298 (19.7)	182 625 (20.5)	18 269 (23.9)	20 089 (21.5)	6307 (24.0)	6195 (21.9)
Gout	28 758 (3.5)	36 815 (4.6)	32 522 (3.6)	42 184 (4.7)	3764 (4.9)	5369 (5.7)	1267 (4.8)	1650 (5.8)
Lupus	1798 (0.2)	1538 (0.2)	1996 (0.2)	1738 (0.2)	198 (0.3)	200 (0.2)	61 (0.2)	58 (0.2)
Parathyroid disorders	3417 (0.4)	3341 (0.4)	4044 (0.5)	3877 (0.4)	627 (0.8)	536 (0.6)	226 (0.9)	169 (0.6)
Thyroid disorders	12 209 (1.5)	10 797 (1.4)	13 596 (1.5)	12 075 (1.4)	1387 (1.8)	1278 (1.4)	477 (1.8)	382 (1.4)
End‐stage renal disease	7040 (0.9)	7689 (1.0)	8136 (0.9)	8793 (1.0)	1096 (1.4)	1104 (1.2)	336 (1.3)	333 (1.2)
Trauma/injuries in the past 90 days	6880 (0.8)	7103 (0.9)	7772 (0.9)	8243 (0.9)	892 (1.2)	1140 (1.2)	325 (1.2)	376 (1.3)
Medication use in the past 6 months, *N* (%)								
Corticosteroid	66 426 (8.1)	80 440 (10.1)	72 386 (8.1)	88 341 (9.9)	5960 (7.8)	7901 (8.4)	2152 (8.2)	2632 (9.3)
Bisphosphonate	33 405 (4.1)	29 879 (3.7)	35 899 (4.0)	32 292 (3.6)	2494 (3.3)	2412 (2.6)	808 (3.1)	695 (2.5)
No of GP active consultation in the past year								
Median (IQR)	8 (4–13)	8 (3–14)	8 (4–14)	8 (4–15)	13 (7–21)	11 (5–20)	15 (9–24)	14 (7–23)
Min, Max	0, 334	0, 364	0, 334	0, 364	0, 248	0, 213	0, 248	0, 208
≥ 12 visits	259 699 (31.7)	267 215 (33.5)	301 190 (33.6)	311 854 (35.0)	41 490 (54.2)	44 638 (47.7)	16 636 (63.2)	16 240 (57.5)
< 12 visits	512 160 (62.6)	477 487 (59.8)	546 137 (61.0)	523 519 (58.7)	33 976 (44.4)	46 031 (49.2)	9486 (36.0)	11 524 (40.8)
None	46 810 (5.7)	53 720 (6.7)	47 902 (5.4)	56 595 (6.3)	1092 (1.4)	2875 (3.1)	197 (0.7)	491 (1.7)
No of hospital admission in the past year								
Median (IQR)	0 (0–1)	0 (0–1)	0 (0–1)	0 (0–1)	0 (0–1)	0 (0–1)	0 (0–1)	0 (0–1)
Min, Max	0, 267	0, 206	0, 267	0, 206	0, 159	0, 204	0, 158	0, 204
≥ 12 admissions	3661 (0.4)	5253 (0.7)	4195 (0.5)	6025 (0.7)	534 (0.7)	772 (0.8)	172 (0.7)	228 (0.8)
< 12 admissions	262 290 (32.0)	260 381 (32.6)	290 157 (32.4)	291 970 (32.7)	27 865 (36.4)	31 587 (33.8)	9581 (36.4)	9768 (34.6)
None	552 718 (67.5)	532 788 (66.7)	600 877 (67.1)	593 973 (66.6)	48 159 (62.9)	61 185 (65.4)	16 566 (62.9)	18 259 (64.6)

*Note:* *Until 31/3/2023 due to limited linked data availability in the main analysis.

Abbreviations: IQR, interquartile range; GP, general practice; NA, not applicable.

Comparing the baseline characteristics in different periods, the distribution of covariates between exposure groups was similar pre‐, during or post‐pandemic lockdowns. However, fluoroquinolone users had marginally fewer comorbidities and were less likely to have ≥ 12 GP active consultations versus cephalosporin users during/post‐pandemic period but not in the pre‐pandemic period. The standardised differences of covariates were slightly larger in Analysis 4 than in other Analyses. However, all the standardised differences between groups were < 0.1 regardless of pandemic periods, indicating good covariate balance (Figure S3). Figure S4 shows the temporal trend of outcome in the study cohort. There was an increasing trend of incidence of first tendon rupture diagnosis in the study cohort throughout the study period with a small drop in 2021. The incidence of outcome in the fluoroquinolone group did not considerably change since the COVID‐19 pandemic but the number of tendon ruptures was very small (< 5) in 2021 and 2022 in the cephalosporin group. Most of the tendon ruptures occurred in ankle and foot (44.1%), followed by shoulder region (34.2%) (Table S1).

In Analysis 1 which uses pre‐pandemic data only, PS‐weighted HR for tendon rupture was 1.94 (95% CI: 1.46–2.59) with a crude RD of 0.77 (95% CI: 0.53–1.02) per 1000 person‐year, comparing fluoroquinolones with cephalosporin, consistent with the previously published study (Table 2). In Analyses 2 and 3, which incorporated data from the pandemic period, results were largely similar to Analysis 1 (Table 2). In Analysis 3, which uses data across all time periods (Table 3), the PS‐weighted HR for tendon rupture was 1.96 (95% CI: 1.47–2.61) for pre‐pandemic period and 1.57 (95% CI: 0.72–3.41) with a wide CI for during/post‐pandemic period. The PS‐weighted HR of the period‐treatment interaction was 0.80 (95% CI: 0.35–1.84) with a wide CI due to low number of tendon rupture for the during/post‐pandemic period. The PS‐weighted HRs for tendon rupture comparing fluoroquinolones with cephalosporin were 1.96 (95% CI: 1.47–2.61) in the pre‐pandemic period and 1.57 (95% CI: 0.72–3.41) during/post‐pandemic period. In Analysis 4, which uses post‐pandemic data only (Table 2), the PS‐weighted HR was 3.32 (95%: 0.62–17.72) with a wide CI due to low number of tendon rupture.

**TABLE 2:** Details of results from univariable and propensity score models in four main analyses.

	Number of events	Total person‐year	Absolute risk per 1000 patients	Rate per 1000 person‐years	Crude rate difference	Unadjusted		Propensity score weighted	
						HR	95% CI	HR	95% CI
Analysis 1									
Cephalosporin	82	129 996	0.10	0.63		1.00 (ref)		1 (ref)	
Fluoroquinolone	179	127 470	0.22	1.40	0.77 (0.53–1.02)	2.23	1.71–2.89	1.94	1.46–2.59
Analysis 2									
Cephalosporin	82	130 259	0.10	0.63		1.00 (ref)		1 (ref)	
Fluoroquinolone	179	127 878	0.22	1.40	0.77 (0.52–1.02)	2.22	1.71–2.89	1.94	1.46–2.58
Analysis 3^a^									
Cephalosporin	93	142 003	0.10	0.65		1.00 (ref)		1 (ref)	
Fluoroquinolone	202	142 520	0.23	1.42	0.76 (0.53–1.00)	2.16	1.69–2.77	1.91	1.46–2.51
Analysis 4									
Cephalosporin	< 5	3820	*	*		1.00 (ref)		1 (ref)	
Fluoroquinolone	6	4148	0.21	1.45	*	2.77	0.56–13.75	3.32	0.62–17.72

*Note:* * redacted due to count < 5.

^a^ The treatment estimates from Analysis 3 were estimated in the univariable and propensity score model without an interaction term. The estimates in the models with an interaction term were reported in Table 3. All estimates are corrected to 2 d.p.

**TABLE 3:** Details of results from univariable and propensity score models in Analysis 3 using all data in study period of 1 January 2006–31 March 2023 with an interaction term in the outcome model.

Treatment estimate FQ vs. cephalosporin	Unadjusted		Propensity score weighted			
	HR	95% CI	HR	95% CI	Interaction HR*	95% CI*
Pre‐pandemic	2.23	1.71–2.89	1.96	1.47–2.61	0.80	0.35–1.84
During/post‐ pandemic	1.67	0.81–3.42	1.57	0.72–3.41		

During/post‐ pandemic vs. pre‐pandemic	Unadjusted		Propensity score weighted			
	HR	95% CI	HR	95% CI	Interaction HR*	95% CI*
Cephalosporin	1.45	0.77–2.72	1.31	0.66–2.58	0.80	0.35–1.84
Fluoroquinolones	1.09	0.71–1.68	1.05	0.65–1.68		

*Note:* * Propensity score weighted interaction HR between treatment and period with 95% CI.

The rates of people who lost follow‐up due to deregistering from their GP (around 52 per 1000 patient‐year) and switching to another treatment group (around 151 per 1000 patient‐year) were low (Table S2). The rates of cephalosporin users lost to follow‐up due to deregistering from their GP and switching to another treatment group were slightly higher than fluoroquinolone users in all analyses, particularly in the post‐pandemic period.

### 3.1 Sensitivity Analyses

Results were similar using CPRD only in a longer study period (i.e., 1 January 2006–31 December 2024) with PS‐weighted HR for period‐treatment interaction of 1.08 (0.50–2.33) and PS‐weighted HR for tendon rupture comparing fluoroquinolones with cephalosporin shifted away from null in the post‐pandemic period in Analysis 3 (HR: 2.01; 95% CI: 1.00–4.05) (Tables S3 and S4).

When we varied the study start year from 2006 to 2019, PS‐weighted HRs varied between 1.52 (95% CI: 1.08–2.13) and 2.47 (95%: 1.18–5.17), with small variations of PS‐weighted RDs between 0.55 (95% CI: 0.13–0.96) and 1.01 (95% CI: 0.25–1.76) per 1000 person‐year (Figures S5 and S6).

## 4 Discussion

Despite the disruption to healthcare caused by the COVID‐19 pandemic, the annual incidence of first tendon rupture diagnosis in this cohort remained broadly stable, with year‐to‐year variation during and after the pandemic similar to that observed before the pandemic. The fluoroquinolones users had more comorbidities than cephalosporin users in the pre‐pandemic period but fewer comorbidities than cephalosporin users in the post‐pandemic period, indicating that the practice of antibiotic prescribing could be different since the COVID‐19 pandemic. Notably, there was a shortage of penicillin after a spike in scarlet fever and group A streptococcal infections in December 2022 [20] and cefalexin was one of the drug alternatives indicated in the Serious Shortage Protocol [21]. This might partly explain the shift in patient characteristics of cephalosporin group during pandemic around December 2022.

However, our study showed that combining pre‐, during and post‐pandemic data resulted in similar findings to those obtained using only data from the pre‐pandemic period in this case study. This applied to both relative and absolute measures of effect, though this is likely to be partly a consequence of relatively few patients and outcomes in the during/post‐COVID‐19 period. Importantly, whichever way we structured the follow‐up period, the results we obtained for the effect of fluoroquinolones compared to cephalosporin on tendon rupture would have led to the same general conclusions.

Although we found substantively similar findings combining all data, we observed an inflated HR with a wide CI in the analysis varying the study start year to 2018 and in the analysis using post‐pandemic data only, though this is due to a relatively small sample size during/post‐COVID‐19 period. We also observed a very small number of events in 2021 and 2022 in the cephalosporin group but not in the fluoroquinolone group. Therefore, the increased HR could also be partly explained by more diligent recording of tendon rupture diagnoses or greater health‐seeking behaviour among fluoroquinolone users than cephalosporin users during COVID‐19 pandemic as tendon rupture is a well‐recognised adverse drug reaction of fluoroquinolones with prominent safety warnings issued by international medicines regulators since 2008 [22, 23]. This reporting bias would be most likely to affect clinical settings where clinicians and patients are aware of a potential association between the exposed drug of interest and effectiveness/safety outcome, and the outcome is not serious leading to hospitalisation and death. Although this type of selective reporting was also present in the pre‐pandemic period, it may be more pronounced during/post‐pandemic due to changes in health‐seeking behaviours.

## 5 Strengths and Limitations

This case study contributes to the limited evidence base on the impact of COVID‐19 pandemic on estimating causal effects. We successfully replicated the previous cohort study [13] and obtained consistent results using pre‐pandemic data only, validating our methodological approach. We also used different settings (i.e., different study periods), presenting results with/without period‐treatment interaction to explore the potential impact of COVID‐19 pandemic lockdown on estimating treatment effects.

This study has some limitations. First, the start dates of during/post‐pandemic period were defined by COVID‐19 stringency Index but the patterns of health‐seeking behaviour and diagnostic recording may vary across different time points with various degrees of restrictions. However, defining finer time periods within during/post‐pandemic was not feasible due to small counts. Second, it is not possible to determine whether changes in diagnoses during the pandemic were due to recording and/or true changes in incidence of some conditions. Tendon rupture associated with fluoroquinolones is a relatively rare outcome. This meant the number of cases occurring during and post‐pandemic was small and this may partially explain why the choice of study analysis period had little impact on the results. Whether this would generalise to studies with more common outcomes is unknown. Moreover, drug adherence and persistence were unknown, leading to potential misclassification bias of drug exposure. However, assuming a non‐differential misclassification of exposure, estimates would be biased towards the null. Finally, although we included some frailty‐related covariates in the PS model, other unmeasured confounding including frailty may remain.

## 6 Implications for Research

Future studies addressing causal questions with follow‐up that spans the COVID‐19 pandemic should first report temporal trends of incidence of outcomes recording within their study cohort and each exposure group. This would allow assessment of whether pandemic lockdown may have affected the recording of that particular outcome and whether the impact was differential between exposure groups before, during and post‐pandemic as the first step. Second, consideration of mechanisms underlying differential outcome ascertainment between the exposure groups during/post‐pandemic is important to interpret findings. For example, when the outcome is not critical, diagnoses might be under‐recorded in the healthcare database due to reduced healthcare utilisation and differences in health‐seeking behaviour. Importantly, outcomes of clinical concern may be more actively detected and recorded in one drug exposure group than in another, particularly for drugs with regulatory safety warnings or heightened public concern. These differences are likely to be more pronounced during/post‐pandemic when disruptions to routine care and uneven monitoring may exacerbate differential disease ascertainment. A period‐treatment interaction can be incorporated to estimate treatment effects separately for pre‐, during/after pandemic periods. This approach allows the investigation of any observed differences in treatment estimates between periods, but as seen here, may have low power for rare outcomes. Alongside investigating diagnostic trends between exposure groups, further investigation may be needed to understand observed differences between periods and whether they arise from measurement error in the outcome or other factors, such as changes to treatments, drug shortages (not only the supply of the drugs of interest but also their drug alternatives) and care received since the COVID‐19 pandemic. We also recommend future research to investigate other types of outcomes, and longer post‐pandemic periods.

In conclusion, combining pre‐, during and post‐pandemic data did not meaningfully change effect estimates in this case study relative to results from the pre‐pandemic period, although information during/post‐pandemic was limited. Future studies with follow‐up that spans the COVID‐19 pandemic should report temporal trends of outcome incidences in each exposure group. Results using during/post‐pandemic data only should be interpreted cautiously, particularly when outcomes may be recorded differently in exposure groups during/post‐pandemic. Future research with a longer study period is also recommended to assess whether the HR based on pre‐pandemic data remains consistent with that based on post‐pandemic data.

## Author Contributions

Conceptualization: A.Y.S.W., J.T., I.D. and C.W.‐G. Data curation: A.Y.S.W. Formal analysis: A.Y.S.W. and J.P.B. Funding acquisition: A.Y.S.W., I.D., C.W.‐G., J.T., J.B. and E.W. Methodology: A.Y.S.W., J.T., M.B., E.W., J.B. and I.D. Writing – original draft: A.Y.S.W. All authors were involved in design, reviewed and approved the final manuscript.

## Funding

This work was supported by the Medical Research Council (MR/Z503769/1). E.W. is funded by a Wellcome Senior Research Fellowship (224485/Z/21/Z). C.W.G. is funded by a Wellcome Career Development Award (225868/Z/22/Z). This research was funded in part by Wellcome.

## Disclosure

For the purpose of Open Access, the author has applied a CC BY public copyright licence to any Author Accepted Manuscript (AAM) version arising from this submission. Funders had no role in the study design, collection, analysis and interpretation of data; in the writing of the report and in the decision to submit the article for publication.

## Ethics Statement

The UK study protocol was approved by the London School of Hygiene and Tropical Medicine Ethics Committee (31308) and the Independent Scientific Advisory Committee for the Medicines and Healthcare products Regulatory Agency (No. 24_004655).

## Consent

The authors have nothing to report.

## Conflicts of Interest

I.J.D. has received research grants from NIHR, GlaxoSmithKline (GSK) and AstraZeneca and holds shares in GSK. A.Y.S.W. received honoraria from National Capacity Building Programme for Human Resources for Health—Empowerment of Young Leaders for Healthy China in 2026, Henry Stewart Talks, 6th Annual meeting of the Society for Clinical Epidemiology in Tokyo and 28th Annual Meeting of the Japanese Society for Pharmacoepidemiology in Kyoto in November 2023, outside the submitted work. The other authors declare no conflicts of interest.

## Supporting information


**Figure S1:** Illustration of the cohort design.
**Figure S2:** Flowchart of the inclusion of study participants.
**Figure S3:** Standardised differences of covariate after propensity score weighting.
**Figure S4:** Temporal trend of first tendon rupture diagnosis in the study cohort from 1 January 2006 to 31 December 2022.
**Table S1:** Tendon rupture by anatomical site in Analysis 3.
**Table S2:** Rates of people deregistered from their GP and switching to another treatment group in different analyses.
**Table S3:** Details of results from univariable and propensity score models in four sensitivity analyses using Clinical Practice Research Datalink data only from 1 January 2006 to 31 December 2024.
**Table S4:** Details of results from univariable and propensity score models in Analysis 3 using Clinical Practice Research Datalink data only from 1 January 2006 to 31 December 2024.
**Figure S5:** Results of hazard ratios in the sensitivity analysis varying study start year.
**Figure S6:** Results of crude rate differences in the sensitivity analysis varying study start year.

## Data Availability

Study protocols are available from the corresponding author upon request for the purposes of reproducing the results. Codelists and analytical codes are available in: https://doi.org/10.17037/DATA.00005295 and https://github.com/ehr‐lshtm/covid‐causal‐inference‐FQ. The study data cannot be made available to other researchers because of the terms specified in Data Use Agreements.

## References

[1] A. M. Butler , M. Burcu , J. B. Christian , et al., “Noninterventional Studies in the COVID‐19 Era: Methodological Considerations for Study Design and Analysis,” Journal of Clinical Epidemiology 153 (2023): 91–101. 10.1016/j.jclinepi.2022.11.011 PMC9671552 36400263

[2] K. E. Mansfield , R. Mathur , J. Tazare , et al., “Indirect Acute Effects of the COVID‐19 Pandemic on Physical and Mental Health in the UK: A Population‐Based Study,” Lancet Digital Health 3, no. 4 (2021): e217–e230. 10.1016/S2589-7500(21)00017-0 PMC7985613 33612430

[3] J. Bodilsen , P. B. Nielsen , M. Søgaard , et al., “Hospital Admission and Mortality Rates for Non‐Covid Diseases in Denmark During Covid‐19 Pandemic: Nationwide Population Based Cohort Study,” BMJ 373 (2021): n1135. 10.1136/bmj.n1135 PMC8142604 34035000

[4] E. Coma , C. Guiriguet , N. Mora , et al., “Impact of the COVID‐19 Pandemic and Related Control Measures on Cancer Diagnosis in Catalonia: A Time‐Series Analysis of Primary Care Electronic Health Records Covering About Five Million People,” BMJ Open 11, no. 5 (2021): e047567. 10.1136/bmjopen-2020-047567 PMC8130761 34006554

[5] L. A. Kapsner , M. O. Kampf , S. A. Seuchter , et al., “Reduced Rate of Inpatient Hospital Admissions in 18 German University Hospitals During the COVID‐19 Lockdown,” Frontiers in Public Health 8 (2020): 594117. 10.3389/fpubh.2020.594117 PMC7838458 33520914

[6] N. Mora , C. Guiriguet , R. Cantenys , et al., “Cancer Diagnosis in Primary Care After Second Pandemic Year in Catalonia: A Time‐Series Analysis of Primary Care Electronic Health Records Covering About 5 Million People,” Family Practice 40, no. 1 (2023): 183–187. 10.1093/fampra/cmac083 PMC9384533 35861148

[7] S. Nourazari , S. R. Davis , R. Granovsky , et al., “Decreased Hospital Admissions Through Emergency Departments During the COVID‐19 Pandemic,” American Journal of Emergency Medicine 42 (2021): 203–210. 10.1016/j.ajem.2020.11.029 PMC7676321 33279331

[8] E. Oikonomou , K. Aznaouridis , J. Barbetseas , et al., “Hospital Attendance and Admission Trends for Cardiac Diseases During the COVID‐19 Outbreak and Lockdown in Greece,” Public Health 187 (2020): 115–119. 10.1016/j.puhe.2020.08.007 PMC7434308 32949881

[9] L. Østergaard , J. H. Butt , K. Kragholm , et al., “Incidence of Acute Coronary Syndrome During National Lock‐Down: Insights From Nationwide Data During the Coronavirus Disease 2019 (COVID‐19) Pandemic,” American Heart Journal 232 (2021): 146–153. 10.1016/j.ahj.2020.11.004 PMC7644438 33160947

[10] E. Stöhr , A. Aksoy , M. Campbell , et al., “Hospital Admissions During Covid‐19 Lock‐Down in Germany: Differences in Discretionary and Unavoidable Cardiovascular Events,” PLoS One 15, no. 11 (2020): e0242653. 10.1371/journal.pone.0242653 PMC7678984 33216804

[11] J. Vila , J. Capsec , M. Bigoteau , et al., “Impact of the First Lockdown Related to the COVID‐19 Pandemic on Ophthalmic Emergencies in a French University Hospital,” Journal Francais D'ophtalmologie 45, no. 6 (2022): 587–596. 10.1016/j.jfo.2022.02.006 PMC9040442 35597678

[12] E. Vrdoljak , M. P. Balja , Z. Marušić , et al., “COVID‐19 Pandemic Effects on Breast Cancer Diagnosis in Croatia: A Population‐ and Registry‐Based Study,” Oncologist 26, no. 7 (2021): e1156–e1160. 10.1002/onco.13791 PMC8250475 33856084

[13] J. P. Brown , K. Wing , C. Leyrat , et al., “Association Between Fluoroquinolone Use and Hospitalization With Aortic Aneurysm or Aortic Dissection,” JAMA Cardiology 8, no. 9 (2023): 865–870. 10.1001/jamacardio.2023.2418 PMC10433140 37585175

[14] C. Alves , D. Mendes , and F. B. Marques , “Fluoroquinolones and the Risk of Tendon Injury: A Systematic Review and Meta‐Analysis,” European Journal of Clinical Pharmacology 75, no. 10 (2019): 1431–1443. 10.1007/s00228-019-02713-1 31270563

[15] E. Mathieu , H. Ritchie , L. Rodés‐Guirao , et al., “Coronavirus Pandemic (COVID‐19),” 2020, https://ourworldindata.org/covid‐stringency‐index.

[16] S. S. Jick , K. W. Hagberg , R. Persson , et al., “Quality and Completeness of Diagnoses Recorded in the New CPRD Aurum Database: Evaluation of Pulmonary Embolism,” Pharmacoepidemiology and Drug Safety 29, no. 9 (2020): 1134–1140. 10.1002/pds.4996 32222005

[17] F. C. K. Dolk , K. B. Pouwels , D. R. M. Smith , J. V. Robotham , and T. Smieszek , “Antibiotics in Primary Care in England: Which Antibiotics Are Prescribed and for Which Conditions?,” Journal of Antimicrobial Chemotherapy 73, no. 2 (2018): 2–10. 10.1093/jac/dkx504 PMC5890730 29490062

[18] E. J. Williamson and A. Forbes , “Introduction to Propensity Scores,” Respirology 19, no. 5 (2014): 625–635. 10.1111/resp.12312 24889820

[19] T. Sturmer , R. Wyss , R. J. Glynn , and M. A. Brookhart , “Propensity Scores for Confounder Adjustment When Assessing the Effects of Medical Interventions Using Nonexperimental Study Designs,” Journal of Internal Medicine 275, no. 6 (2014): 570–580. 10.1111/joim.12197 PMC4037382 24520806

[20] G. Iacobucci , “Strep A: Pharmacists Can Supply Alternative Penicillin, in Light of Shortages,” BMJ 379 (2022): o3020. 10.1136/bmj.o3020 36691355

[21] NHS Business Services Authority , “Serious Shortage Protocol (SSP) for Phenoxymethylpenicillin 125 mg/5mL Oral Solution Sugar Free—Corresponding Guidance on Endorsement, Prescription Charges, Remuneration and Reimbursement Plus Q&A,” 2026, https://www.nhsbsa.nhs.uk/sites/default/files/2023‐01/240120~2.PDF.

[22] Medicines and Healthcare products Regulatory Agency , “Fluoroquinolone Antibiotics: Reminder of the Risk of Disabling and Potentially Long‐Lasting or Irreversible Side Effects,” 2023, https://www.gov.uk/drug‐safety‐update/fluoroquinolone‐antibiotics‐reminder‐of‐the‐risk‐of‐disabling‐and‐potentially‐long‐lasting‐or‐irreversible‐side‐effects.

[23] J. H. Tanne , “FDA Adds “Black Box” Warning Label to Fluoroquinolone Antibiotics,” BMJ 337, no. 7662 (2008): a816. 10.1136/bmj.a816 PMC2483892 18632714

