# Supplementary material for: Impact of COVID‐19 Restrictions on Estimating Causal Effects: Fluoroquinolones and Tendon Rupture
Source: Pharmacoepidemiol Drug Saf. 2026 Oct 15;35(11):e70504. doi: 10.1002/pds.70504 (PMC13671875; doi:10.1002/pds.70504)
Supplement: Supplementary file 1 [file PDS-35-e70504-s001.docx]

**Supplementary: Impact of COVID-19 restrictions on estimating causal effects: Fluoroquinolones and tendon rupture.**

Contents

[Figure S1. Illustration of the cohort design	2](#_Toc238743641)

[Material S1. Details of the implementation of period-treatment interaction in Analysis 3	3](#_Toc238743642)

[Figure S2. Flowchart of the inclusion of study participants	4](#_Toc238743643)

[Figure S3. Standardised differences of covariate after propensity score weighting	5](#_Toc238743644)

[Figure S4. Temporal trend of first tendon rupture diagnosis in the study cohort from 1/1/2006 to 31/12/2022	6](#_Toc238743645)

[Table S1. Tendon rupture by anatomical site in Analysis 3	7](#_Toc238743646)

[Table S2. Rates of people deregistered from their GP and switching to another treatment group in different analyses	8](#_Toc238743647)

[Table S3. Details of results from univariable and propensity score models in four sensitivity analyses using Clinical Practice Research Datalink data only from 1/1/2006-31/12/2024	9](#_Toc238743648)

[Table S4. Details of results from univariable and propensity score models in Analysis 3 using Clinical Practice Research Datalink data only from 1/1/2006-31/12/2024	10](#_Toc238743649)

[Figure S5. Results of hazard ratios in the sensitivity analysis varying study start year	11](#_Toc238743650)

[Figure S6. Results of crude rate differences in the sensitivity analysis varying study start year	12](#_Toc238743651)

### **Figure S1. Illustration of the cohort design**

### Material S1. Details of the implementation of period-treatment interaction in Analysis 3

In Analysis 3, there were three types of patients:

1. Time zero (i.e. cohort entry) and event/censoring time were both in pre-pandemic period

2. Time zero was in pre-pandemic period while event/censoring time was in post-pandemic period

3. Time zero was in post-pandemic period

The data management for time-varying covariate only affect type 2 patients who had the follow-up spanning the start of COVID-19 pandemic lockdown (i.e. 16^th^ March 2020). A second row for each type 2 patient was therefore created, with this second row corresponding to their post-pandemic follow-up period. A “startdate” variable, which tells Stata what time on the time scale this episode of follow-up started.

Here is an example of the dataset:

| id | exposure | timezero | startdate | period | enddate | event |
| --- | --- | --- | --- | --- | --- | --- |
| 1 | 1 | 23feb2015 | 23feb2015 | 0 | 18Mar2015 | 1 |
| 2 | 1 | 14mar2020 | 14mar2020 | 0 | 16mar2020 | 0 |
| 2 | 1 | 14mar2020 | 16mar2020 | 1 | 31mar2020 | 1 |
| 3 | 0 | 10nov2021 | 10nov2021 | 1 | 23nov2021 | 0 |

Codebook:

id: individual Patient ID

exposure: binary (1=fluoroquinolone, 0=cephalosporin)

timezero: the start of follow-up

startdate: start date of the interval (i.e. time zero or 16^th^ March 2020 if their intervals spanning 16^th^ March)

period: indicates pre- or on/after pandemic lockdown (based on 16 March 2020)

enddate: date of event/end of follow-up if no event

event: binary 1/0 (1=event, 0=censoring)

In this example, Patient id 1 and 3 represents type 1 and 3 patients respectively as their time zero were both in pre-pandemic and post-pandemic periods respectively. Patient id 2 represent type 2 patients as the time zero was in pre-pandemic period (14 March 2020) while the event occurred in the post-pandemic period (31 March 2020). Therefore, a second row beginning the interval from 16^th^ March to 31^st^ March 2020 was created for Patient id 2.

After the data management, the data can be prepared for time-to-event analysis (univariable model) using Stata as follows:
stset enddate id(id) fail(event) enter(startdate) origin(timezero)

The Cox proportional hazard model with period-treatment interaction can then be conducted using Stata as follows:
stcox i.exposure##i.period

### Figure S2. Flowchart of the inclusion of study participants

*210,645 patients were excluded in the propensity score model due to missing data for ethnicity, body mass index, alcohol consumption, smoking status or sex. 19 patients were further excluded due to non-overlapping propensity score distribution. In Analysis 3, the number of fluoroquinolone and cephalosporin users were 786,038 and 790,495 respectively in the propensity score model.

Figure S3. Standardised differences of covariate after propensity score weighting

Figure S4. Temporal trend of first tendon rupture diagnosis in the study cohort from 1/1/2006 to 31/12/2022

*The number of diagnoses in 2023 was not presented as the data for the whole year is not available. The incidence in cephalosporin group in 2021 and 2022 was redacted due to frequency of tendon rupture <5 in each year.

Incidence was calculated as: number of outcomes (in study cohort/ each group) divided by number of individuals per 1000 with active follow-up during that year (in study cohort/ each group)

Table S1. Tendon rupture by anatomical site in Analysis 3

| **Body site** | **N (%)** |
| --- | --- |
| Ankle and Foot | 130 (44.1) |
| Should region | 101 (34.2) |
| Hand | 13 (4.4) |
| Lower leg | 8 (2.7) |
| Pelvic region and thigh | 8 (2.7) |
| Other/ Site unspecified | 35 (11.9) |

### Table S2. **Rates of people** **deregistered from their GP and switching to another treatment group in different analyses**

| Rates of deregistered from GP per 1000 patient year | Fluoroquinolone | Cephalosporin |
| --- | --- | --- |
| Analysis 1 | 43.4 | 57.7 |
| Analysis 2 | 43.3 | 57.6 |
| Analysis 3 | 44.0 | 59.8 |
| Analysis 4 | 47.6 | 78.4 |
| Rates of switching to another treatment group per 1000 patient year |  |  |
| Analysis 1 | 146.1 | 165.3 |
| Analysis 2 | 145.8 | 165.1 |
| Analysis 3 | 138.7 | 162.7 |
| Analysis 4 | 77.4 | 123.8 |

### Table S3. **Details of results** from univariable and propensity score models in four sensitivity analyses using Clinical Practice Research Datalink data only from 1/1/2006-31/12/2024

|  | | Number of events | | | Total person-year | Rate per 1,000 | | | Crude  rate difference | | | Univariable  model | | | | Propensity score  model | | |
| --- | --- | --- | --- | --- | --- | --- | --- | --- | --- | --- | --- | --- | --- | --- | --- | --- | --- | --- |
|  | | |  | |  | |  | |  | | HR | | | 95% CI | | HR | 95% CI | |
| **Analysis 1** |  | | |  | | | |  | |  | | |  | |  | | |  |
| Cephalosporin | | | 78 | | 130,060 | | 0.60 | |  | | 1.00 (ref) | | |  | | 1 (ref) |  | |
| Fluoroquinolone | | | 166 | | 127,519 | | 1.30 | | 0.70 (0.46-0.94) | | 2.17 | | | 1.66 - 2.84 | | 1.85 | 1.33 - 2.56 | |
| **Analysis 2** |  | | |  | | | |  | |  | | |  | |  | | |  |
| Cephalosporin | | | 78 | | 130,322 | | 0.60 | |  | | 1.00 (ref) | | |  | | 1 (ref) |  | |
| Fluoroquinolone | | | 166 | | 127,927 | | 1.30 | | 0.70 (0.46-0.94) | | 2.17 | | | 1.66 - 2.84 | | 1.84 | 1.33 - 2.55 | |
| **Analysis 3*** | |  | | |  |  | | |  | | |  | | | |  | | |
| Cephalosporin | | | 91 | | 148,122 | | 0.61 | |  | | 1.00 (ref) | | |  | | 1 (ref) |  | |
| Fluoroquinolone | | | 198 | | 148,302 | | 1.34 | | 0.72 (0.50-0.95) | | 2.17 | | | 1.70 - 2.79 | | 1.89 | 1.41 - 2.55 | |
| **Analysis 4^a^** |  | | |  | | | |  | |  | | |  | |  | | |  |
| Cephalosporin | | | 5 | | 9875 | | 0.51 | |  | | 1.00 (ref) | | |  | | 1 (ref) |  | |
| Fluoroquinolone | | | 15 | | 9882 | | 1.52 | | 1.01 (0.12-1.90) | | 3.01 | | | 1.09 - 8.28 | | 3.34 | 1.13 – 9.83 | |

*The treatment estimates from Analysis 3 were estimated in the univariable and propensity score model without an interaction term. The estimates in the models with an interaction term were reported in Table S4. All estimates are corrected to 2 d.p.

### Table S4. **Details of results** from univariable and propensity score models in Analysis 3 using Clinical Practice Research Datalink data only from 1/1/2006-31/12/2024

|  | **Unadjusted** | | **Propensity score weighted** | | | |
| --- | --- | --- | --- | --- | --- | --- |
| **Treatment estimate**  **FQ vs. cephalosporin** | | | | | **Interaction between treatment and period** | |
|  | HR | 95% CI | HR | 95% CI | HR | 95% CI |
| **Pre-pandemic** | 2.17 | 1.66 - 2.84 | 1.87 | 1.35 - 2.59 | 1.08 |  |
| **During/post- pandemic** | 2.14 | 1.12 – 4.08 | 2.01 | 1.00 – 4.05 |  | 0.50 – 2.33 |
|  | **Unadjusted** | | **Propensity score weighted** | | | |
| **During /post-pandemic vs. pre-pandemic** | | | | | **Interaction between treatment and period** | |
|  | HR | 95% CI | HR | 95% CI | HR | 95% CI |
| Cephalosporin | 1.20 | 0.67 – 2.16 | 1.13 | 0.60 – 2.15 | 1.08 |  |
| Fluoroquinolones | 1.18 | 0.81 – 1.73 | 1.22 | 0.79 – 1.88 |  | 0.50 – 2.33 |

Figure S5. Results of hazard ratios in the sensitivity analysis varying study start year

*Abbreviation: PS, propensity score; CI, confidence interval; HR, hazard ratio; py, person-year

Figure S6. Results of crude rate differences in the sensitivity analysis varying study start year

*Abbreviation: PS, propensity score; CI, confidence interval; RD, rate difference; py, person-year
